# Detection and characterization of translational research in cancer and cardiovascular medicine

**DOI:** 10.1186/1479-5876-9-57

**Published:** 2011-05-11

**Authors:** David S Jones, Alberto Cambrosio, Andrei Mogoutov

**Affiliations:** 1 Program in Science, Technology, and Society, Massachusetts Institute of Technology, Cambridge, MA, USA; 2 Department of Global Health and Social Medicine, Harvard Medical School, Boston, MA, USA; 3 Department of Social Studies of Medicine, McGill University, Montreal, Canada; 4 Institut Francilien Recherche Innovation Société, Marne la Vallée, France

## Abstract

**Background:**

Scientists and experts in science policy have become increasingly interested in strengthening translational research. Efforts to understand the nature of translational research and monitor policy interventions face an obstacle: how can translational research be defined in order to facilitate analysis of it? We describe methods of scientometric analysis that can do this.

**Methods:**

We downloaded bibliographic and citation data from all articles published in 2009 in the 75 leading journals in cancer and in cardiovascular medicine (roughly 15,000 articles for each field). We calculated citation relationships between journals and between articles and we extracted the most prevalent natural language concepts.

**Results:**

Network analysis and mapping revealed polarization between basic and clinical research, but with translational links between these poles. The structure of the translational research in cancer and cardiac medicine is, however, quite different. In the cancer literature the translational interface is composed of different techniques (e.g., gene expression analysis) that are used across the various subspecialties (e.g., specific tumor types) within cancer research and medicine. In the cardiac literature, the clinical problems are more disparate (i.e., from congenital anomalies to coronary artery disease); although no distinctive translational interface links these fields, translational research does occur in certain subdomains, especially in research on atherosclerosis and hypertension.

**Conclusions:**

These techniques can be used to monitor the continuing evolution of translational research in medicine and the impact of interventions designed to enhance it.

## Background

The past decade has seen unprecedented interest in translational medicine. Many experts have recommended strategies to overcome the "valley of death" that separates basic science from its practical applications[1-7]. Federal agencies, professional societies, and research centers can all provide dedicated funding, incentives for translational research, infrastructure that supports dialogue across disciplinary divides, and better integration of clinical research into both basic science and health care delivery[1,5,8-10]. If policy interventions are going to be designed and implemented, policy makers need to know where translational research is happening, and why, so that they can formulate and test policy innovations that might foster it. Unfortunately, defining translational medicine and assessing its impact has been difficult[1].

New research emerging at the intersection of sociology and computer science offers tools that can help achieve these goals[11]. Computer techniques can analyze large datasets of publications and citations in order to characterize and map the structure of scientific fields and their development over time,[12,13] and even to model the dissemination of scientific ideas and identify characteristics of publication patterns that suggest whether an idea or innovation has reached a crucial phase transition[14,15]. Medical researchers have made increasing use of these techniques recently. One team used citation analysis to track stages in the development of angioplasty research [16-18]. Another researcher analyzed citation networks to study the prevalence of the belief in a relationship between β-amyloid and Alzheimer's Disease, showing for instance that researchers often failed to cite papers that did not support the model[19]. A third group looked at breast cancer research from 1945 to 2008, focusing on research output by country; countries with higher rates of international research cooperation produced papers that were more likely to be highly cited[20]. Another group has looked more broadly at cancer publications and studied the impact of funding and public policy on cancer research [21-23]. This work shows that scientometric techniques can be a powerful way to reveal patterns in medical research and publishing that would not be evident with traditional methods of reviewing the medical literature.

Our group has adapted these methods to study translational gaps. More specifically, we have used three different tools -- inter-citation, co-citation and semantic network analysis -- to investigate the emergence, structure, and content of translational research in biomedicine by comparing research in cancer and cardiovascular medicine.

Journal inter-citation is the relation established when an article in Journal A cites an article in Journal B. Analysis of inter-citation patterns reveals how closely journals are related based on the journals cited by articles that they publish. A network map of inter-citation connections provides an overall view of the knowledge structure of a field and its subfields[24-26]. Clusters within the networks can be further characterized by determining the *research level *of their constitutive journals. As developed by Lewison and Paraje,[27] research level avoids the simple split between basic and applied science by rating journals on a continuous scale according to keywords in the titles of the articles they publish, and then using thresholds to divide the journals into a four-fold scheme: clinical observation, clinical mix, clinical research, and basic research. This can reveal at a glance whether and how clinical and research journals refer to each other. Our initial work, focused on cancer research, documented the emergence of a translational interface between 1980 and 2000[28]. Specifically, inter-citation analysis in 1980 revealed two distinct domains, one focused on basic science and one on clinical oncology. By 2000 a distinct third domain had appeared. It had strong internal cross-links, suggesting that it had its own questions and methods. But it occupied an intermediate position between basic and clinical science and had strong links to each, suggesting that it bridged those two poles. It had the features of a translational interface.

Journal inter-citation only shows links between journals without providing information about actual content of those journals. This is especially a problem with generalist or multi-disciplinary journals whose content spans a wide range of topics. Semantic network analysis can fill in this gap by probing the actual content of publications. Multi-term concepts are extracted from journal titles and abstracts using text mining software with natural language processing algorithms. The resultant network of co-occurring terms can be displayed as a map, along with the journals in which they most frequently appear. The map, with its linked clusters, can reveal the content of translational interfaces.

A third technique can reveal the historical development of these interfaces. Article A and article B are co-cited if they appear together in the reference list of a subsequent article; the assumption is that co-cited articles are related and of relevance to researchers in that particular domain at that point in time. Maps of the most frequently co-cited articles reveal what researchers see as the key contributions to their field and can therefore display the cognitive substructure of a field[24-26]. Mapping co-citation along a temporal axis can demonstrate the contribution of both older and more recent articles to the formation of a given specialty or domain. This does not show the actual historical development of a field; instead, it reveals judgments about the relevant history of a field as perceived at a given moment in time (i.e., as perceived by the authors of the articles used as source data).

In this paper we extend our previous work by comparing the structure of the translational interface in oncology and cardiovascular medicine in 2009. Each domain is a major component of contemporary biomedicine. But this does not necessarily mean that the structure and substance of translational research in these areas follows the same or similar patterns.

## Methods

### Publication Data

We used *Journal Citation Report *(2008 edition) to identify the leading journals in cancer and cardiovascular medicine. For cancer, we used JCR's "Oncology" category. For cardiovascular medicine, we combined "Cardiac & Cardiovascular Medicine" and "Peripheral Vascular Disease." For each field we selected the seventy-five journals that published the most articles. We then downloaded, from *ISI Web of Science*, bibliographic data on every article in those journals in 2009; we excluded reviews, editorials, letters, and other document types. This produced a set of 18,581 articles for cancer and 15,421 articles for cardiovascular medicine.

### Analyses

These downloads provided the data required for three distinct sets of analyses.

(1) Journals: Inter-citation analyses can be performed between articles or between journals. Since we were interested in relationships between journals, and not in the relationships between articles, we aggregated the articles into their respective journals and then determined journal-journal links based on the citations from all articles in a given journal to the other journals. Some journals are citing journals (i.e., journals from which we downloaded articles), some journals are cited journals (i.e., journals we did not select, but that appear in the citations, especially generalist journalists like the *New England Journal of Medicine *or *JAMA*), and some are both cited and citing (i.e., *Circulation*).

Once data have been obtained, the analyses can be performed with many network analysis software packages; we used *ReseauLu *(http://www.aguidel.com), which has algorithms designed specifically to import bibliographic data and perform scientometric analyses of heterogeneous networks (i.e., networks that include different date types, such as journals, keywords, authors, genes, proteins, diseases, or any other category, depending on the data available)[29]. The analyses require two distinct steps.

First, we established which journals had significant inter-citation relationships. Because of the density of connections (i.e., tens of thousands of citations), it is not possible to map every link between every journal. To produce a legible map it is necessary to discern the most relevant links between journals. We did this with a Chi Square specificity measure[28,30]. This measure is calculated by creating a two-dimensional array, with rows corresponding to citing journals and columns corresponding to cited journals. Each cell of this array contains the actual number of citations from one journal to another, the observed value (OV). We defined the marginal frequency (MF) of each citing journal as the sum of the observed values in its row, and the marginal frequency of each cited journal as the sum of the observed values in its column. The total number of citations is the sum of all observed values in the array. The actual observed distribution can be compared to a null hypothesis in which the occurrences of the values in the array (e.g., the journal inter-citations) are statistically independent. The expected value (EV) for each cell in this null hypothesis is defined as follows:

The specificity of the relationship between a cited and citing journal is then simply the standardized residual (SR), the value of the deviance of a cell's observed value from its expected value:

We set a threshold and kept only the subset of cells having the highest standardized residual. In this case, based on empirical assessment of the resulting maps, we arbitrarily set a threshold of the top 15% most specific links; this achieved a useful balance of connectivity and legibility. We treated this as a binary variable: Journal X either did or did not have a specific link to each other journal.

Second, once we had determined which journals did have specific citation links, we used *ReseauLu's *dynamic positioning algorithm to map inter-citation relationships between the journals. This algorithm models each journal as an object connected to other objects by springs. The spring was either rigid or elastic, depending on whether or not a specific link existed. The dynamic positioning algorithm optimized the position of all of the nodes in order to minimize the overall strain in the network[28]. Either one of two extreme conditions -- all nodes equally connected to each other, or no nodes connected at all -- will produce a homogeneous and symmetrical distribution within a circular space. Data sets between these two extremes will yield maps that have clusters of nodes that reflect the relationships between the mapped objects. The proximity of two journals is not directly representative of the specific strength of relationship between them, but instead represents the overall set of relationships of that journal and the other journals to which it is specifically linked.

To facilitate interpretation of the network plots, we color-coded the title of each journal according to its *Research Level*. This is an independent measure developed by Lewison and Paraje,[27] unrelated to our own analyses. Research Level rates journals as clinical observation (color-coded as blue), clinical mix (green), clinical research (orange), and basic research (red), based on analysis of the titles of articles published in each journal. This color-coding makes it easier to distinguish journals focused on clinical care from those focused on basic research. We also highlighted relevant clusters that occur in the maps, whether clusters of journals by research level or clusters of journals by topic. While network analysis algorithms can be used to automate the identification of clusters,[31] for our purposes here visual inspection and subjective assessment can reliably identify the most obvious clusters.

(2) Keywords: We used natural language processing (NLP) algorithms to extract the 250 most prevalent multi-word concepts from the titles and abstracts from all articles in the top 75 journals in each field. As we have described in detail elsewhere, one approach to NLP uses hard-coded dictionaries and a sequence of morphological, syntactic, semantic, pragmatic, and statistical treatments in order to recognize parts of speech, to examine relationships between terms, to resolve ambiguities, and to select candidate single- and multi-word concepts[31]. The rapidly increasing sophistication of NLP algorithms over recent years has improved the reliability and utility of this approach. Compared to other approaches, such as analyzing the co-occurrence of the MeSH keywords used to index articles listed in the PubMed database, NLP has several advantages. Most importantly, it provides access to the concepts actually used by the authors, instead of relying on the standardized vocabulary imposed by the MeSH indexers[32]. Such use of standardized vocabularies can blur the textual specificity of each article[33].

We performed these analyses with SPSS Lexiquest Mine (now available as IBM SPSS Modeler and IBM SPSS Text Analytics); other packages can presumably perform comparable analyses. We then constructed a heterogeneous map of the 20 most publishing journals and the 250 most prevalent concepts. We began by establishing whether significant relationships existed within the bipartite graph of journals and concepts: using the Chi Square specificity measure described above, we calculated the weighted discrepancy between the observed and expected (based on a null hypothesis of independent distribution) number of occurrences of a concept in the articles published in a journal. In this case, however, we set the specificity threshold at 30% to produce a legible map. We then mapped the links using the dynamic positioning algorithm described above.

(3) Key Articles: For both cancer and cardiac medicine we used network analysis software to select the 100 articles most often co-cited by the articles in each set of specialty journals. Here, instead of selecting and mapping some portion of the most specific links, we mapped only the links between the nearest nodes, as follows. The strength of association between any two nodes (i.e., between Article X and Y) is calculated as the number of links between those two nodes (i.e., the number of times X and Y were co-cited) divided by the square root of the product of the frequency of X and the frequency of Y in the overall dataset. For each article, we kept the five links with the highest value -- the five "nearest nodes." This measure is not necessarily symmetric: Article X might have Article Y as one of its nearest nodes, but not vice versa. The choice of the measure of relevance (e.g., most specific links vs. nearest nodes) is an arbitrary empirical choice. In our experience, the nearest nodes algorithm produces the most legible maps for co-citation networks[31]. For these maps, we added a historical perspective. After the dynamic positioning algorithm had run and established the best distribution of the nodes, the resultant network was stretched onto a temporal axis so that the oldest nodes appear at the top, and the more recent ones along the bottom. We then examined the distribution of nodes, and the articles represented by each, to identify clusters of articles on specific topics. This helps to reveal the historical development of leading subfields, as perceived from 2009.

Additional File 1 lists the top 75 articles in each field and the top 250 concepts from the article subsets, and provides further information about the 100 most co-cited articles (authors, journal, title) which is needed to interpret the co-citation maps.

## Results and Discussion

### Journal Inter-Citation

**Figure 1:**
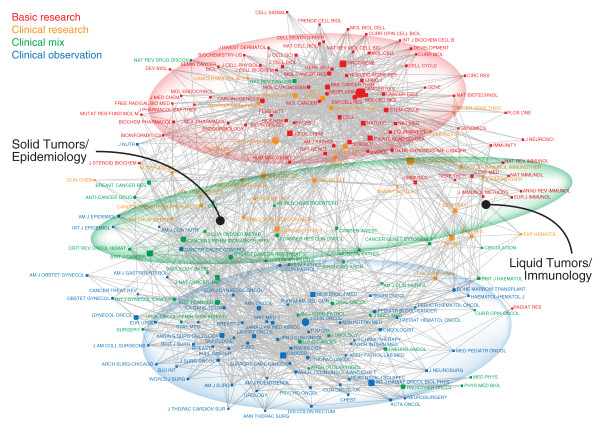
**Journal-Journal Inter-Citation Network of the Cancer Literature in 2009**. Each node represents a specific journal. The size of the node indicates the prominence of the journal in the literature (specifically, number of articles published in 2009). The map shows the 75 journal in our dataset (the citing journals, shown with circular nodes, e.g. the *Journal of Clinical Oncology*) and the 200 most cited journals (shown with square nodes if they are not in the citing journal set, e.g., the *New England Journal of Medicine*). Each line reflects an inter-citation relationship between the two journals. To increase legibility, only the 15% most specific links are included. Tightly connected journals appear close to each other. Tightly linked sub-networks can be seen by the dense web of connections among them. The nodes and journal names are color-coded according to research levels: blue for clinical observation, green for clinical mix, orange for clinical research, and red for basic research. Journals dedicated to basic science and molecular biology cluster at the top, with *Cancer Research *and *Oncogene *forming the largest nodes. Journals focused on clinical topics, such as the *Journal of Clinical Oncology *and *Cancer *cluster at the bottom. Between the two poles can be found a translational interface of clinical research and clinical mix journals. Journals focused on solid tumors are to the left (e.g., *Breast Cancer Research*, *Prostate*, *Gastroenterology*), and journals focused on hematologic tumors are on the right (e.g., *Blood*, *Leukemia*).

The cancer journals in 2009 exhibit the same basic patterns we had seen in 2000. The journals segregate into two distinct poles, one focused on basic research, the other on clinical observation (Figure 1). A band of clinical research and clinical mix journals lies between the two poles, with journals dedicated to solid and hematologic tumors segregated within this. This distinct translational interface exists as its own domain, with strong cross-links to each pole. This network is not linked to a specific function or subspecialty within cancer research (e.g., breast cancer or lung cancer or leukemia), but instead reflects allegiance to a common orientation of research work. It spans the full range of clinical problems in cancer, from solid tumors to liquid tumors, often involving specific techniques (e.g. gene expression analysis) that are useful across all cancer types.

**Figure 2:**
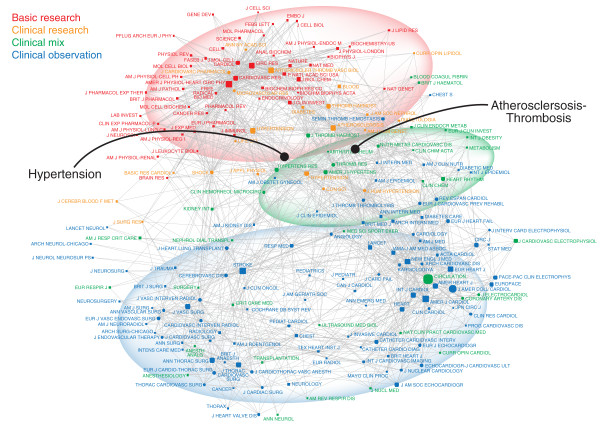
**Journal-Journal Inter-Citation Network of the Cardiac Literature in 2009**. The network map was prepared as described for Figure 1. The clinical pole, along the bottom, dominates the figure, with distinct clusters: stroke and neurology on the left, cardiac surgery on the bottom, imaging on the bottom right, and then on the right the largest domain, focused on clinical cardiology, centered around the *Journal of the American College of Cardiology*, the *American Journal of Cardiology*, and the *European Heart Journal*. The basic research pole is confined to the upper left, anchored by *Circulation Research *and the *American Journal of Physiology - Heart and Circulatory Physiology*. Dense connections do exist between the clinical and basic science poles, but these are focused on journals on two topics: atherosclerosis (from *Atherosclerosis *to *Arteriosclerosis Thrombosis and Vascular Biology*) and hypertension (from *American Journal of Hypertension *to *Hypertension *and on into basic research in pharmacology).

The map of the cardiac journal inter-citation shares some of these features (Figure 2). Basic research and clinical research clusters are located at the top left. Clinical observation journals dominate the lower half, with a split between surgery on the left and cardiology and internal medicine on the right. The clinical mix journals occupy an intermediate position, suggestive of a translational interface, but they are more intermingled with the clinical journals. *Circulation*, for instance, the largest node of the clinical mix journals, is positioned squarely among the journals of clinical cardiology. Some caution is needed here. *Circulation *is a diverse journal that publishes a wide range of articles. Although it exists at a single location on the map, it encompasses everything from clinical reports to clinically relevant findings of basic research.

Important differences exist, however, between this and the cancer map. First, there is a striking preponderance of clinical articles and journals in the cardiac literature: clinical journals dominate a larger area of the cardiac map than the cancer map. Of the 75 most active cardiac journals, a higher percentage have a clinical focus than in the corresponding set of cancer journals. With such a small presence of basic science journals, the basic research pole is actually formed of both the basic and clinical research journals. There is less intellectual distance, in some respects, between the extremes of the cardiac literature. In contrast to the cancer literature, where the most basic and most clinical cancer journals are fundamentally distinct, there is more common content across the cardiac journals.

Second, although links do exist between basic research and clinical research poles, a distinct translational interface has not yet appeared in cardiovascular medicine. While topics such as hypertension and atherosclerosis do have links between the clinical and research poles, they do not form a coherent third domain. The maps show that translational research is taking place in each field, but with important differences in the translational interfaces. There are many possible causes of this. In the case of cancer, despite the existence of different subspecialties defined by the anatomic site of the cancer (e.g., lung, breast, colon, etc.), there may be a translational interface defined by specific approaches (e.g., gene expression profiling) that are used across all cancer types. In cardiovascular medicine, in contrast, there appear to be distinct clinical domains (e.g., atherosclerosis, hypertension), each with its own translational links. There appears to be no distinct space in the cardiac domain for the kind of broad-reaching translational research seen in the cancer domain. Possible hypotheses can be assessed by evaluating the semantic content of the interfaces.

### Semantic Structure

**Figure 3:**
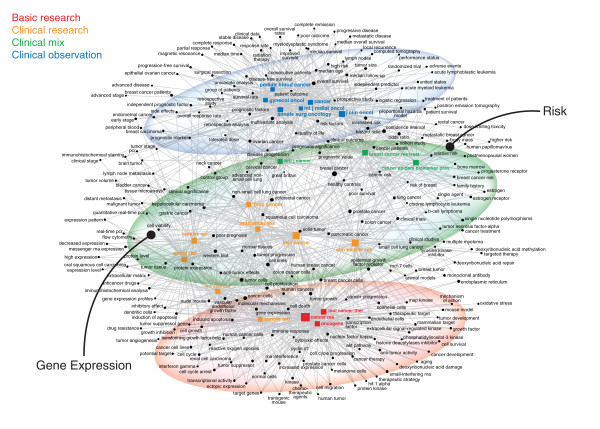
**Journal-Concept Co-Occurrence Map for the Cancer Literature in 2009**. The 20 most publishing journals (square nodes, colored according to research level) and the 250 most prevalent single- and multi-word concepts are mapped according to the strength of association between concept and journals; only the 30% most specific links are mapped. The map has three distinct zones. Clinical journals (e.g., *Journal of Clinical Oncology, Cancer, Annals of Surgical Oncology*) cluster at the top, with links to terms related to epidemiology and clinical trials (e.g., high risk, quality of life, overall survival, univariate analysis). Basic science and clinical research journals (e.g., *Oncogene, Cancer Research*) cluster at the bottom, with links to terms related to molecular biology and genetics (e.g., transcription factor, kinase, immune response, therapeutic target). The translational interface (from *Cancer Science *to *Cancer Epidemiology*) includes two sets of terms. One set reflects specific tumor types (e.g., prostate cancer, breast cancer, colorectal cancer.). The other set reflects specific translational techniques, with gene expression technologies on the left (e.g., PCR, western blot, microarrays) linked to clinical research journals (in orange) and ideas linked to the polysemic notion of risk on the right (e.g., cancer risk, family history, poor survival) linked to clinical mix journals (in green).

**Figure 4:**
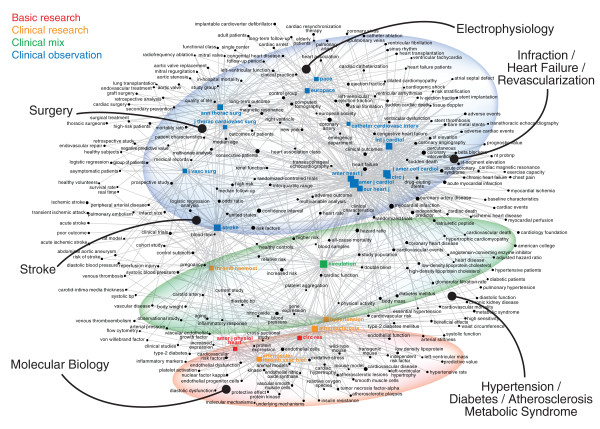
**Journal-Concept Co-Occurrence Map for the Cardiac Literature in 2009**. The network map was prepared as described for Figure 3. Clinical observation journals and concepts dominate the top half of the map. Distinct clusters can be seen for stroke on the left, surgery and electrophysiology on the top, and myocardial infarction, heart failure, and treatments (e.g., angioplasty) on the right. A small cluster of terms from molecular biology (e.g., protein kinase, nuclear factor kappa, tumor necrosis factor) exists at the bottom, linked to *Circulation Research*, *American Journal of Physiology - Heart*, and *Atherosclerosis, Thrombosis, and Vascular Biology*. No clearly structured translational interface exists. *Circulation*, however, the sole clinical mix journal in the set, maintains links to both the clinical domain and the molecular biology domain. This journal plays a key role linking diverse research interests.

Similar structures appear in the maps of the semantic content of the two fields. The cancer map shows three distinct zones (Figure 3). Clinical journals are situated at the top, linked to terms about clinical trials and epidemiology. Journals focused on genetics and cancer biology cluster at the bottom, linked to the language of molecular biology. A translational interface exists between these poles, with several sets of concepts. One set, appearing in clinical research journals, involves gene expression technologies that are used for diagnosis, prognosis, and clinical research. Another, in the clinical mix journals, involves concepts of risk and the tools used to assess it. Clinical journals and concepts dominate the cardiac map, with clinical observation journals filling the top half (Figure 4). Clinical research and basic research journals and their associated concepts from molecular biology appear only on the periphery at the bottom. The translational interface is less clear, in part because there is only one clinical mix journal (*Circulation*), four clinical research, and two basic research journals in the set (compared to three, eight, and three for cancer). This reflects a significant difference between the two fields. When we selected journals, we concentrated on specialty journals in cancer and cardiac medicine. While the cancer literature does have a broad range of journals, from clinical (e.g., *Cancer*) to basic (e.g., *Oncogene*), the specialty journals in cardiac medicine are more focused on clinical problems and methods. The research pole that does exist in the cardiac semantic map contains a mix of the language of clinical science (e.g., risk factors, biomarkers) and molecular biology (e.g., protein kinase, endothelial progenitor cells).

### Article Co-Citation

**Figure 5:**
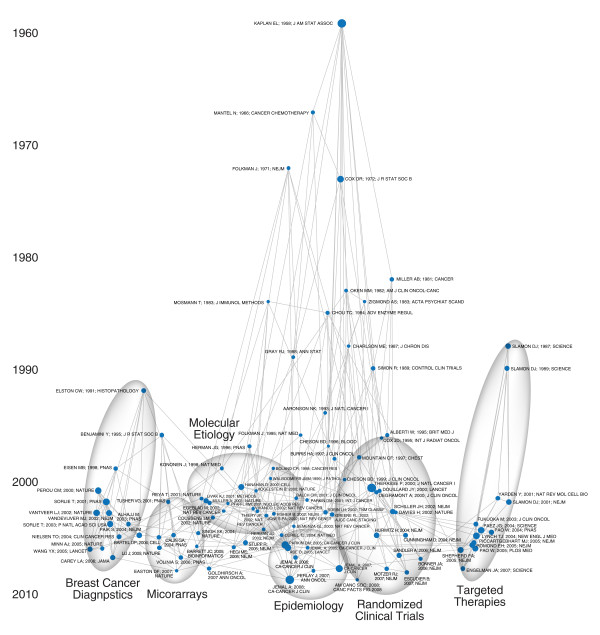
**Article-Article Co-Citation Network of the Cancer Literature in 2009**. Co-citation relationships of the 100 most co-cited articles are mapped. The articles are arrayed along a chronological axis according to their publication date. The links between them show the co-citation relationships made by articles published in 2009 (i.e., this is a view of the field from the vantage point of 2009). Sheet 3A in Additional File 1 lists the articles plotted here. Circles were added to highlight specific clusters. The central portion of the plot is dominated by articles about cancer clinical trials (Schiller 2002, Hurwitz 2004, Cunningham 2004), looking back to the articles about relevant statistical methods (Kaplan Meier 1958, Mantel 1966, Cox 1972). The largest recent node here (Therasse 2000) provides guidelines about assessing treatment response. The cluster also includes papers on cancer staging (e.g., TNM classifications -- Sobin 2002). An adjacent cluster focuses on epidemiology (Jemal 2006, Jemal 2007, Jemal 2008). The next cluster to the left, reaching back to early work on angiogenesis (Folkman 1971), now includes articles on the molecular etiology of cancer, including oncogenes, P53, HF1, AKT pathways, and HPV (Hanahan 2000, Vogelstein 2000, Vivanco 2002). Towards the left can be found papers on PCR, tissue microarrays, and iRNA (Tusher 2001, Lu 2005). Thus there is an overlap between the topic (molecular etiology) and the techniques needed to study it. The cluster on the far left focuses on cancer diagnostics (classification, prognosis, and prediction), from early papers on histopathological grading (Elston 1991), to the key papers on the molecular biology and genomic signatures of breast cancer (Perou 2000, Sorlie 2001, van't Veer 2002, Paik 2004). These overlap with articles on the bioinformatic methods needed to analyze microarrays and similar genomic tools (Benjamini 1995, Eisen 1998, Tusher 2001). A distinct cluster on the far right includes research on specific receptors and the new targeted therapies, starting from the landmark papers on HER2 (Slamon 1987) and extending through more recent work on HER2, EGFR, and the new drug that target those receptors, including Herceptin/Trastuzumab and Iressa/Gefitinib (e.g., Slamon 2001, Fukuoka 2003, Lynch 2004, Pao 2004, Shepherd 2005, Engeman 2007). The bottom of the plot thus reveals a continuum from work on the molecular biology, especially of breast cancer, on the left to clinical trials of targeted therapies on the right, through research on molecular pathways and RCTs, and related statistical methods.

**Figure 6:**
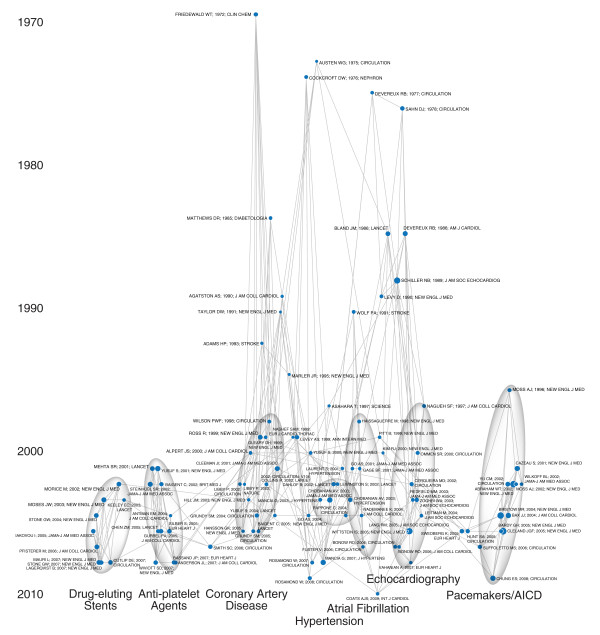
**Article-Article Co-Citation Network of the Cardiac Literature in 2009**. The network map was prepared as described for Figure 5. Sheet 3B in the Additional File lists the articles published here. The most recent articles are divided into distinct clusters by clinical topic. On the far left are articles about drug-eluting stents (from Morice 2002 to Stone 2007), adjacent to another thin cluster about clopidogrel and anti-platelet agents (Yusuf 2001, Mehta 2001, Wivott 2007). The central cluster has concentrations of articles about cholesterol, atherosclerosis, and myocardial infarction (Wilson 1998, Libby 2004, Yusuf 2004, Hansson 2005), then hypertension (Dahlof 2002, Chobanian 2003, Mancia 2007), and atrial fibrillation (Haissaguerre 1998, Go 2001, Pappine 2004, Fuster 2006). The right edge of the cluster has articles about echocardiography (Nagueh 1997, Ommen 2000, Lang 2005). Finally, at the extreme right of the map sit articles about implanted defibrillators and biventricular pacing (Moss 1997, Bristow 2004, Bardy 2005, Chung 2008). As was seen in the cancer map, the most enduring articles all involve specific clinical or laboratory techniques, such as measurement of LDL (Friedeweld 1972) or creatinine clearance (Cockcroft 1976), or standards, whether for grading coronary artery disease (Austen 1975) or quantifying echocardiography (Devereux 1977, Schiller 1989).

The article co-citation plots reveal the structure of the field as visualized by links between articles that seemed relevant in 2009. The cancer plot has two basic components (Figure 5). The oldest articles, at the top, describe fundamental statistical techniques, especially biostatistical methods used in clinical trials, that remain relevant for research today. The bottom of the plot shows areas of active research. These cluster in informative ways. The left side includes articles on the molecular biology of cancer. The right side focuses on new targeted therapies. The middle is composed of articles on clinical trials. The cardiac map shows a different structure (Figure 6). The recent articles are organized not by research technique but by clinical topic, from drug-eluting stents on the bottom left to automatic implantable cardiac defibrillators and pacemakers on the right. This is consistent with the basic structure of the cardiac field, as revealed by both journal inter-citation and semantic analysis.

It is worth noting that the most cited articles in both lists include several different types of articles. Some are research articles. Others, especially the older ones, are descriptions of widely used methods and techniques. One interesting set are the guidelines and criteria of various sorts seen in both the cancer plot (Boland 1998, Mountain 1997, Therasse 2000, Sobin 2002) and the cardiac plot (Schiller 1989, Chobanian 2003, Lang 2005, Mancia 2007). The prominence of such guidelines demonstrates the importance of standardization and regulatory tools for both research and clinical care[34].

Citation analysis also reveals evidence of ritual use of citations. It is likely that many of the recent articles that cite the oldest articles (e.g., Kaplan Meier 1958) do so without having read the classic articles. For instance, while 267 of the 2009 articles correctly cited this article (Kaplan and Meier, *Journal of the American Statistical Association*, 1958), 170 -- nearly 40% of the citers -- cited it incorrectly (*Journal of the American Medical Association*). The problem of citation mutation has received increasing attention recently[35].

## Conclusions

Our findings demonstrate that systematic analysis of publication and citation data from tens of thousands of articles can capture important features of active fields of scientific research, in this case in both cancer and cardiac medicine. The relational maps based on this data are well structured, stable over time, and accessible to interpretation. They reveal at a glance a polarization between clinical and basic research, as well as the structures that connect these poles. They also reveal clear differences in the relationships of journals in oncology and cardiac medicine.

Do these differences arise from the nature of the clinical problems and the research they require? For most of the late twentieth century, cancer was seen as a problem of cellular pathology, with research focused on the cellular and molecular basis of the disease. These methods and concepts could be applied to most cancers, regardless of their cell of origin. Cardiac researchers, in contrast, focused longer on problems of organ pathology and physiology (e.g., valve disease, coronary occlusion, arrhythmias). Only in specific areas -- notably the biology of hypertension and atherosclerosis -- did molecular biology take root early, and these are exactly the areas where the translational domain is clearest.

The maps also identify areas where the connections are not as strong. Surgical research, in both cancer and cardiac care, is on the periphery of the plots, less strongly connected to translational and basic research than other areas in the fields. On the cardiac map some links do appear between the surgical journals and *Shock*; surgical oncology journals may also be linked to molecular biology through adjuvant and neo-adjuvant trials. Areas where links are less clear suggest possible targets for research investment. The recent increase in interest in efforts to engineer and grow vascular tissues for use as conduits in surgical reconstruction, for instance, may eventually fill this gap in the cardiac literature.

The distinct structures in cardiac and cancer publishing require explanation. Are the differences intrinsic to the science of the field itself, or are they produced by institutional structures, policy initiatives, or clinical concerns? Developments in cancer research in the 1990s, including rapid advances in understanding of cancer genetics and increasing use of surface antigens to characterize cell types, yielded a hybrid of applied and basic research with clear relevance for clinical oncology. Cardiac therapeutics, in contrast, have remained less unified, with clusters of clinicians and associated researchers working on distinct topics such as coronary revascularization or management of hypertension, heart failure, or arrhythmias. It may be that the clinical problems in cardiac medicine are so distinctive that no broad translational interface should be expected. But this also raises important questions. Does the current structure of each field reflect the internal cognitive or epistemological content of the field? Or does the current structure reflect past policy decisions that directed resources to certain kinds of clinical and research questions? Presumably both sets of factors contribute. Further insight into these questions could be gained through a range of different bibliometric analyses. We analyzed and presented data at a high level of aggregation (i.e., by journals or by articles). Other analyses could be performed, for instance based on the institutional affiliations of authors, the countries where research takes place, or on co-authorship relationships. We looked briefly at some of these analyses but found the ones described in detail here to be more fruitful.

Assessing the relative importance of substantive content and policy initiative for the structure of scientific fields has consequences for the prospects of current policy decisions. Policy makers today have argued that by providing funding, incentives, and infrastructure support it will be possible to integrate basic and clinical research and foster translational research[1,3,6,7,10]. This history provides a test case. Were there differences in research policy between cancer and cardiovascular research that account for the different outcomes in those two fields? Further analysis of these questions is needed to provide a firm baseline for policy interventions that seek to overcome the translational divides that exist in medical research today.

Even though we cannot offer definitive explanations for why the structures and differences exist -- experts in the respective fields will have to fill in this history and derive appropriate policies -- we do offer a set of relevant parameters and tools that can be used to study the on-going efforts to strengthen translational research in health care.

## Competing interests

We are not aware of any conflicts of interest relevant to this paper. AM is a co-founder of Aguidel, the company that produces ReseauLu. Although we used this software for our analyses, other software packages (as we state explicitly) could also be used.

## Authors' contributions

All authors participated in the design of the study. AC and DJ acquired the bibliographic datasets. AC conducted the keyword extraction. AM performed the analyses and prepared the maps. DJ drafted the initial manuscript. All authors contributed to the revisions and approved the final manuscript.

## Supplementary Material

Additional file 1 **Specific Information about Journals, Keywords, and Articles**. This is an xls spreadsheet that contains five separate sheets. Sheets 1A and 1B list the top 75 journals (by number of articles published) in cancer and cardiac medicine, selected with data from *Journal Citation Reports *(using the 2008 edition), including the number of articles and impact factor of each journal in 2009. Sheet 2 lists the top 250 most frequently occurring concepts in the cancer and cardiac literature, extracted from the articles in the top 75 journals in each field for 2009. This data is mapped in Figures 3 and 4. Sheets 3A and 3B list the 100 most frequently co-cited articles in the cancer and cardiac literatures (i.e., from all articles published in the top 75 journals in 2009). This provides fuller bibliographic data for the articles shown in Figures 5 and 6. Click here for file
